# Notes and Notices

**Published:** 1899-08

**Authors:** 


### NOTES AND NOTICES.

Chateau de Speer.—See the Chateau de Speer on another page where Alfred Speer, the most honest and persevering wine grower in this country, forty-eight years persistent in overcoming obstacles and prejudices against native wines, has succeeded in New Jersey, and now produces the finest wines of the world and has his extensive wine cellars with hundreds of thousand gallons stored. They are most excellent.

Dr. Joseph T. O'Connor will remove September 1st, 1899, from 18 West Forty-third Street to 29 West Forty-fifth Street, New York, Borough of Manhattan. Office hours till 12 M., 6 to 7 P. M.

For Sickness Get the Best.—Old Choice Wines from Speer's vineyards. The rich Port, Claret, Burgundy and Unfermented are unexcelled for entertainments, family use and invalids.

Wine for Weakly Persons.—Weakly persons use Speer's Port Grape Wine, unfermented Grape Juice and Burgundy or Claret. They give tone and strength to the system. They are superior to all other wines in the world.

